# Preoperative prediction of cavernous sinus invasion by pituitary adenomas using a radiomics method based on magnetic resonance images

**DOI:** 10.1007/s00330-018-5725-3

**Published:** 2018-09-25

**Authors:** Jianxing Niu, Shuaitong Zhang, Shunchang Ma, Jinfu Diao, Wenjianlong Zhou, Jie Tian, Yali Zang, Wang Jia

**Affiliations:** 1 Neurosurgery, Beijing Tiantan Hospital, Capital Medical University, Beijing, 100050 China; 2 CAS Key Laboratory of Molecular Imaging, Institute of Automation, Beijing, 100190 China; 3 University of Chinese Academy of Sciences, Beijing, 100080 China; 4 CAS Center for Excellence in Brain Science and Intelligence Technology, Institute of Automation, Chinese Academy of Sciences, Beijing, 100190 China

**Keywords:** Pituitary adenomas, Cavernous sinus, Neoplasm invasion, Nomogram, Support vector machine

## Abstract

**Objectives:**

To predict cavernous sinus (CS) invasion by pituitary adenomas (PAs) pre-operatively using a radiomics method based on contrast-enhanced T1 (CE-T1) and T2-weighted magnetic resonance (MR) imaging.

**Methods:**

A total of 194 patients with Knosp grade two and three PAs (training set: *n* = 97; test set: *n* = 97) were enrolled in this retrospective study. From CE-T1 and T2 MR images, 2553 quantitative imaging features were extracted. To select the most informative features, least absolute shrinkage and selection operator (LASSO) was performed. Subsequently, a linear support vector machine (SVM) was used to fit the predictive model. Furthermore, a nomogram was constructed by incorporating clinico-radiological risk factors and radiomics signature, and the clinical usefulness of the nomogram was validated using decision curve analysis (DCA).

**Results:**

Three imaging features were selected in the training set, based on which the radiomics model yielded area under the curve (AUC) values of 0.852 and 0.826 for the training and test sets. The nomogram based on the radiomics signature and the clinico-radiological risk factors yielded an AUC of 0.899 in the training set and 0.871 in the test set.

**Conclusions:**

The nomogram developed in this study might aid neurosurgeons in the pre-operative prediction of CS invasion by Knosp grade two and three PAs, which might contribute to creating surgical strategies.

**Key Points:**

• *Pre-operative diagnosis of CS invasion by PAs might affect creating surgical strategies*

• *MRI might help for diagnosis of CS invasion by PAs before surgery*

• *Radiomics might improve the CS invasion detection by MR images.*

**Electronic supplementary material:**

The online version of this article (10.1007/s00330-018-5725-3) contains supplementary material, which is available to authorized users.

## Introduction

Pituitary adenomas (PAs) are common intracranial tumours [1]. Although considered benign, 25–55% of PAs are invasive since they invade adjacent tissues, such as the diaphragma sellae, sphenoid sinus, and cavernous sinus (CS), which corresponds to a more aggressive biological behaviour [1–3].

Surgical removal is the first-line treatment for most pituitary macro-adenomas [4, 5]. However, when planning for surgical removal, CS invasion has been a serious concern [6]. For PAs with CS invasion, combining incomplete removal and neo-adjuvant radiotherapy is recommended, because complete removal is very difficult and can easily injure the trunk and branches of the internal carotid artery (ICA) [7–9]. For PAs without CS invasion, complete removal is recommended, because incomplete removal may lead to a low rate of endocrinological remission and a high rate of recurrence [10–12]. Thus, the preoperative prediction of CS invasion by PAs might aid the surgical strategy making and allow a more focused and cost-effective follow-up and long-term management. The gold standard relies on intraoperative findings—the perforation of the CS medial wall or CS dural involvement by PAs, through which neurosurgeons can distinguish compression from invasion of CS [10, 13]. Currently, Knosp grade is used to evaluate the extent of parasellar extension by PAs before surgery [14]. It was confirmed that CS invasion occurred in all PAs with Knosp grade four and no PAs with Knosp grade zero and one; however, the preoperative diagnosis of CS invasion remained uncertain in Knosp grade two and three PAs [15]. Thus, this study focused on Knosp grade two and three PAs. As CS invasion reflects the morphological relationship between PAs and the CS and MR images can well distinguish tissue structure in the sellar region [10, 13], we hypothesised that quantitative MR imaging features can improve the evaluation of CS invasion by Knosp grade two and three PAs, and attempted to predict the CS invasion by these PAs before surgery.

To this aim, radiomics, which has emerged in the field of medical imaging analysis in recent years, is a reasonable approach. It transforms a medical image into a large number of quantitative imaging features and then analyses these features using a series of machine learning algorithms [16–18]. Radiomics has been used in the diagnosis or prognosis of colorectal cancer, non-small-cell lung cancer, and gliomas [19–24]. All these studies suggest that radiomics is useful in the analysis of medical images. Through this non-invasive radiomics approach, we aimed to predict CS invasion by Knosp grade two and three PAs before surgery.

## Patients and methods

### Patients

**Fig. 1:**
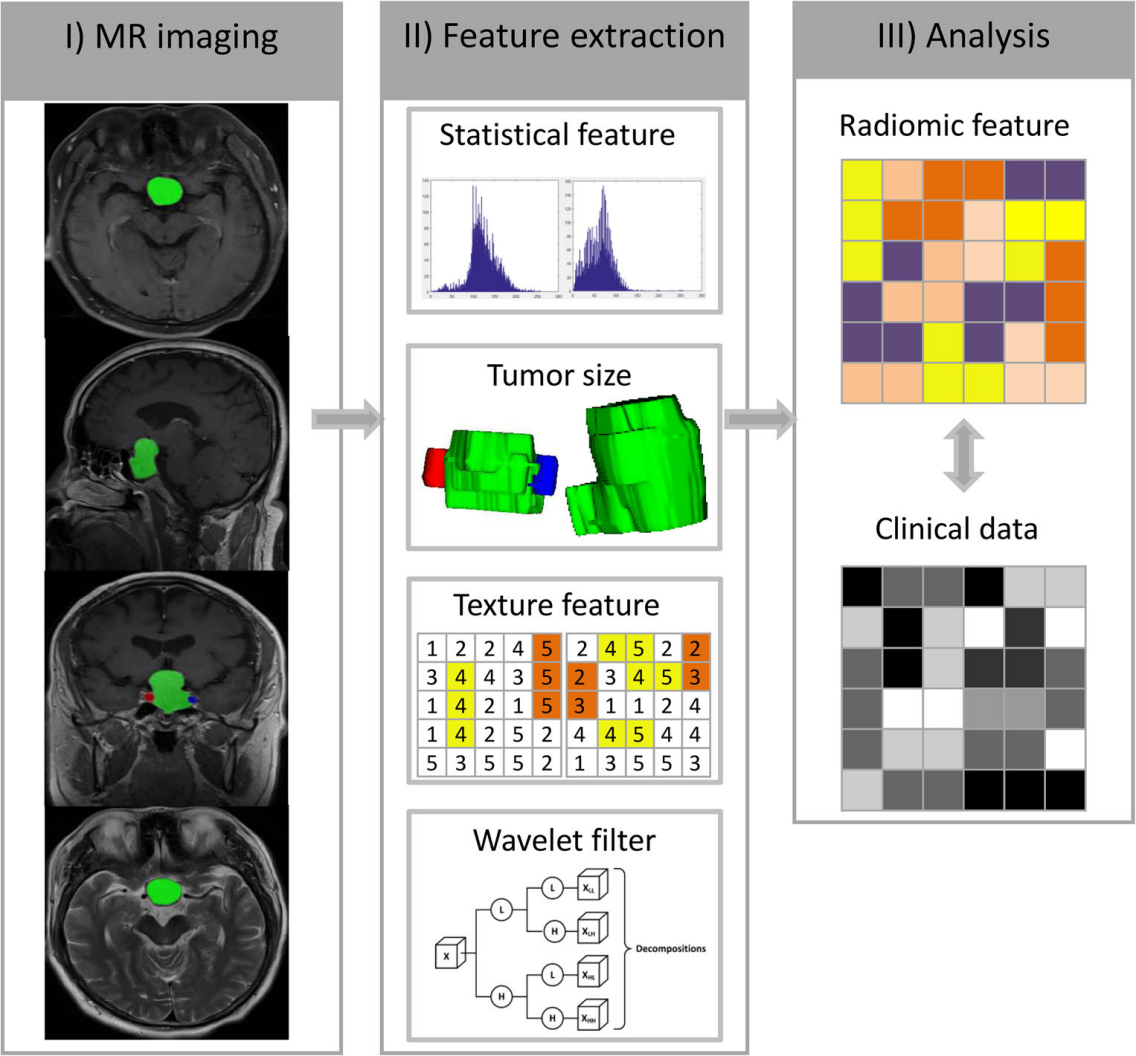
Flowchart illustrating the process of radiomics. I) Image segmentation was conducted on the axial, sagittal and coronal planes of contrast-enhanced T1-weighted MR images and T2-weighted MR images. Note that internal carotid artery on the coronal planes was also contoured. II) Features were extracted from the tumour region. III) Analysis of the radiomics features and clinical data

Ethical approval was obtained for this retrospective analysis from the Institutional Review Board of Beijing Tiantan Hospital Affiliated to Capital Medical University, and the need for informed consent was waived. All patients with pituitary tumour who underwent surgical resection at our institute from July 2013 to July 2016 were enrolled. A radiologist (Reader 1) reviewed the operating records, and another radiologist (Reader 2) reviewed the MR images and assessed the clinico-radiological risk factors (such as Knosp grade, haemorrhage, suprasellar invasion, periarterial enhancement, and inferolateral venous compartment) with no prior knowledge of the operating records. A total of 194 patients (96 men and 98 women; age, 47.02 ± 12.41 years) were identified based on inclusion and exclusion criteria (Supplementary S1). All 194 patients were divided into the training set (*n* = 97, July 2013–July 2014) and the test set (*n* = 97, August 2014–July 2016) according to the MR images’ acquisition time. The training set was used for radiomics signature building, while the test set was set aside for radiomics signature validation. For all cases, tumour resection was performed with the aid of a microscope, and CS invasion status was determined according to the operating records, where the performing neurosurgeons documented their impressions. The Flowchart of this study was shown in Fig. 1.

### Data acquisition

The imaging protocol included unenhanced T1-weighted and T2-weighted images, followed by CE-T1 images. In this study, CE-T1 and T2 MR images were used for analysis. The coronal and sagittal planes of CE-T1 MR images were acquired with repetition time/echo time of 1200/11, acquisition matrix of 256 × 256, and slice thickness of 3 mm; The axial planes of CE-T1 MR images were acquired with repetition time/echo time of 2000/9.8, acquisition matrix of 220 × 185, and slice thickness of 5 mm; T2 MR images were acquired with repetition time/echo time of 4500/84, acquisition matrix of 259 × 384, and slice thickness of 5 mm. Both CE-T1 and T2 MR images are of high resolution on each slice image and make the boundaries of tissues clear; particularly, CE-T1 MR images allow radiologists to distinguish PAs from normal pituitary and surrounding tissues [25]. Furthermore, CE-T1 MR images improve the depiction of CS and ICA due to strong enhancement of the venous compartment after gadolinium-based contrast administration [13]. In addition, CE-T1 and T2 MR images are included in the conventional MR imaging protocol for pituitary macro-adenomas [26]. The acquisition parameters and other details are presented in Supplementary S2.

### Tumour segmentation

Tumour segmentation was conducted on sagittal, coronal, and axial CE-T1 and T2 MR images, and ICA segmentation was conducted on coronal CE-T1 MR images for each patient using the ITK-SNAP program (University of Pennsylvania, www.itksnap.org). The segmentation process was delineated manually by a radiologist (Reader 2) without prior knowledge of the operating records. During tumour segmentation, the slices on which the tumour region was too small (< 10 pixels) were excluded. As the tumour region is usually not as strongly enhanced as surrounding tissues after gadolinium-based contrast administration, pituitary macro-adenomas can be distinguished from surrounding tissues in CE-T1 MR images [25], facilitating tumour segmentation on such images. For T2-weighted MR images, the tumour region was delineated referred to CE-T1 MR images. some representative cases are shown in Fig. S3.

### Feature extraction

A total of 1911 quantitative features describing intra-tumour heterogeneity were extracted automatically from the tumour region on sagittal, coronal, and axial planes of CE-T1 MR images; 641 features were extracted from the tumour region on T2 MR images. These features can be mainly divided into four groups: (I) tumour intensity [27], (II) tumour shape and size, (III) tumour texture features [28–32], and (IV) tumour wavelet features. Aside from these four-group features, another feature describing the degree of PA invasion toward the ICA (ICA wrapped degree) was also calculated based on the ICA region on the coronal planes of CE-T1 MR images. The calculation of all features was implemented in MATLAB 2012a (MathWorks, Natick, MA, USA), and the details of these features are shown in Supplementary S3.

### Statistical analysis

Statistical analysis was performed using MATLAB 2012a. Student’s t-test was used in the analysis of continuous variables, and Pearson’s *χ*^2^ test was used for categorical variables. When a small count existed in the contingency tables, Fisher’s exact test was used instead of Pearson’s *χ*^2^ test. *P*-values were corrected for multiple testing by controlling the false discovery rate of 5%, and two-sided *p*-values < 0.05 were considered statistically significant.

#### Feature selection

High-dimensional data may contain a high degree of redundant and irrelevant information, which can result in overfitting and greatly degrade the performance of the learning algorithm; thus, feature selection is necessary [33]. In this study, feature selection was performed in two stages based on the training set for CE-T1 and T2 MR imaging features, respectively. To explore the advantage of combining CE-T1 and T2 MR imaging features, such features were concatenated, and then the same two-stage feature selection was performed. First, redundant imaging features were removed when the linear correlation coefficient was > 0.75. The least absolute shrinkage and selection operator (LASSO) algorithm [34] was then applied to select the most representative features. A five-fold cross-validation was performed to select the best *λ*—a parameter in LASSO to be determined—using 1-SE criteria; thus, representative features were chosen. The details of LASSO were shown in Supplementary S4. The feature selection procedure was implemented in MATLAB 2012a using a function called *lasso*. ICA wrapped degree was thought to be a representative feature and was not included in the two-stage feature selection.

#### Radiomics model development and validation

Having obtained the representative features, support vector machine (SVM) was used in the training set to build the models for CE-T1, T2, and CE-T1 and T2 MR images. SVMs have been used in glioma grading [35] and survival prediction [36] and turned out to be useful. In this study, the SVMs were trained using LIBSVM [37] with a linear kernel. The parameter *C* was optimised based on a four-fold cross-validated grid search. Finally, the model was developed in the training set with the parameter *C* chosen. The linear SVM prediction score, which was calculated by the transformation of the representative features, was regarded as the radiomics signature. The performance of the CE-T1, T2, and CE-T1 and T2 models was first assessed in the training set and then validated in the test set using the area under the curve (AUC), accuracy, sensitivity, and specificity. Moreover, the receiver operation characteristic (ROC) [38] curve was plotted to illustrate the predictive performance.

#### Clinico-radiological model development and validation

The clinico-radiological risk factors comprised of gender, age, tumour volume, Knosp grade (2 or 3), tumour diameter, haemorrhage (yes or no), suprasellar invasion (yes or no), periarterial enhancement (yes or no), and inferolateral venous compartment obliteration (yes or no). Univariate analysis was performed to show the relationship between CS invasion and each clinico-radiological risk factor. Multivariate logistic regression analysis was applied to develop a clinico-radiological model for predicting CS invasion by PAs in the training set. Forward stepwise selection was conducted using the likelihood ratio test with Akaike’s information criterion (AIC) [39] as the stopping rule. Subsequently, the test set was used to validate the performance of clinico-radiological model.

#### Development and validation of an individualised nomogram

To provide an individual tool for the clinician and patients to predict CS invasion by PAs, a nomogram [40] incorporating the radiomics signature and clinico-radiological risk factors was constructed in the training set and validated in the test set. The calibration curves were plotted for the training and test sets, and the Hosmer-Lemeshow test was conducted to assess the agreement between the predicted risks and observed outcomes of CS invasion. To assess the clinical usefulness of the nomogram, decision curve analysis (DCA) [41] was performed to quantify the net benefits at different threshold probabilities.

## Results

### Clinical characteristics

A total of 194 patients (age, 47.02 ± 12.41 years) were enrolled in this study, among which 82 patients (42.27%) with PA were found with CS invasion. The characteristics of patients and tumours were shown in Table 1. No significant differences for all clinic-radiological factors (*p* = 0.213–0.830) were found between the training set and test set, which justified their use as training set and test set.

**Table 1:** Characteristics of patients and tumours (*n* = 194)

Characteristic	Training Set (*n* = 97)	Test Set (*n* = 97)	Whole Set (*n* = 194)	*p*-value
Age (yr, mean ± std)	47.82 ± 12.46	46.22 ± 12.37	47.02 ± 12.41	0.559
Gender (No.)				0.727
Male	46 (47.42%)	50 (51.55%)	96 (49.48%)	
Female	51 (52.58%)	47 (48.45%)	98 (50.52%)	
Tumour Volume (cm^3^, mean ± std)	14.03 ± 16.87	11.59 ± 8.76	12.81 ± 13.46	0.378
Knosp Grade (No.)				0.213
Grade 2	37 (38.14%)	52 (53.61%)	89 (45.88%)	
Grade 3	60 (61.86%)	45 (46.39%)	105 (54.12%)	
Haemorrhage (No.)				0.830
Yes	12 (12.37%)	13 (13.40%)	25 (12.89%)	
No	85 (87.63%)	84 (86.60%)	169 (87.11 %)	
Tumour Diameter (cm, mean ± std)	3.27 ± 0.97	3.01 ± 0.83	3.14 ± 0.91	0.213
Suprasellar Invasion (No.)				0.378
Yes	64 (65.98%)	55 (56.70%)	119 (61.34%)	
No	33 (34.02%)	42 (43.30%)	75 (38.66%)	
Periarterial Enhancement (No.)				0.830
Yes	43 (44.33%)	45 (46.39%)	88 (45.36%)	
No	54 (55.67%)	52 (53.61%)	106 (54.64%)	
ICV obliteration (No.)				0.378
Yes	30 (30.93%)	21 (21.65%)	51 (26.29%)	
No	67 (69.07%)	76 (78.35%)	143 (73.71%)	

*P*-values were corrected for multiple testing by controlling the false discovery rate of 5%

*yr year*, *std* standard deviation, *ICV* inferolateral venous compartment

### Feature selection

For CE-T1 MR images, 65 imaging features remained after removing the redundant features (correlation coefficient > 0.75); for T2 MR images, 24 imaging features remained, and for CE-T1 and T2 MR images, 89 imaging features remained. Two representative features (Sphericity and Minimum_HL) were selected for CE-T1 images, one (Sphericity) for T2 images, and three (Sphericity [CE-T1], Minimum_HL [CE-T1], and Low Grey Level Run Emphasis_HH_135° [T2]) for CE-T1 and T2 images using LASSO (Fig. S2, Supplementary S4). Apart from these selected features, ICA wrapped degree calculated based on the coronal planes of CE-T1 MR images, was also selected. These representative features are listed in Table 2.

**Table 2:** The list of representative features selected

MR Image	Selected features
CE-T1 MRI	Sphericity; Minimum_HL; ICA Wrapped Degree
T2 MRI	Sphericity
CE-T1&T2 MRI	Sphericity (CE-T1); Minimum_HL (CE-T1); ICA Wrapped Degree (CE-T1); Low Grey Level Run Emphasis_HH_135° (T2)

ICA Wrapped Degree represented the degree of ICA wrapped by tumours

*CE-T1MRI* contrast-enhanced T1 weighted MR image, *T2 MRI* T2 weighted MR image, *ICA* internal carotid artery

### Radiomics model development and validation

**Fig. 2:**
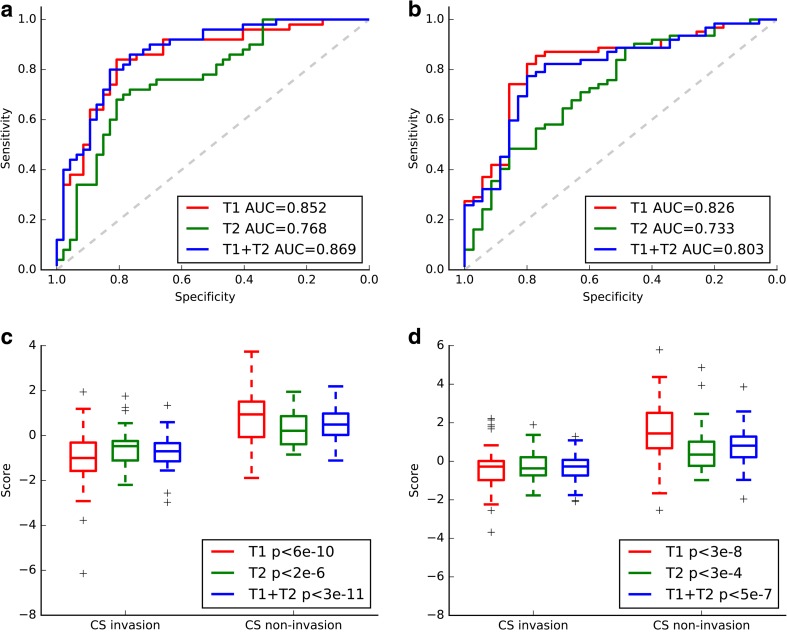
Performance of radiomics models based on CE-T1, T2, and CE-T1 and T2 images. The ROC curves (**a**) and boxplots (**c**) of the three models on the training set. The ROC curves (**b**) and boxplots (**d**) of the three models on the test set

**Fig. 3:**
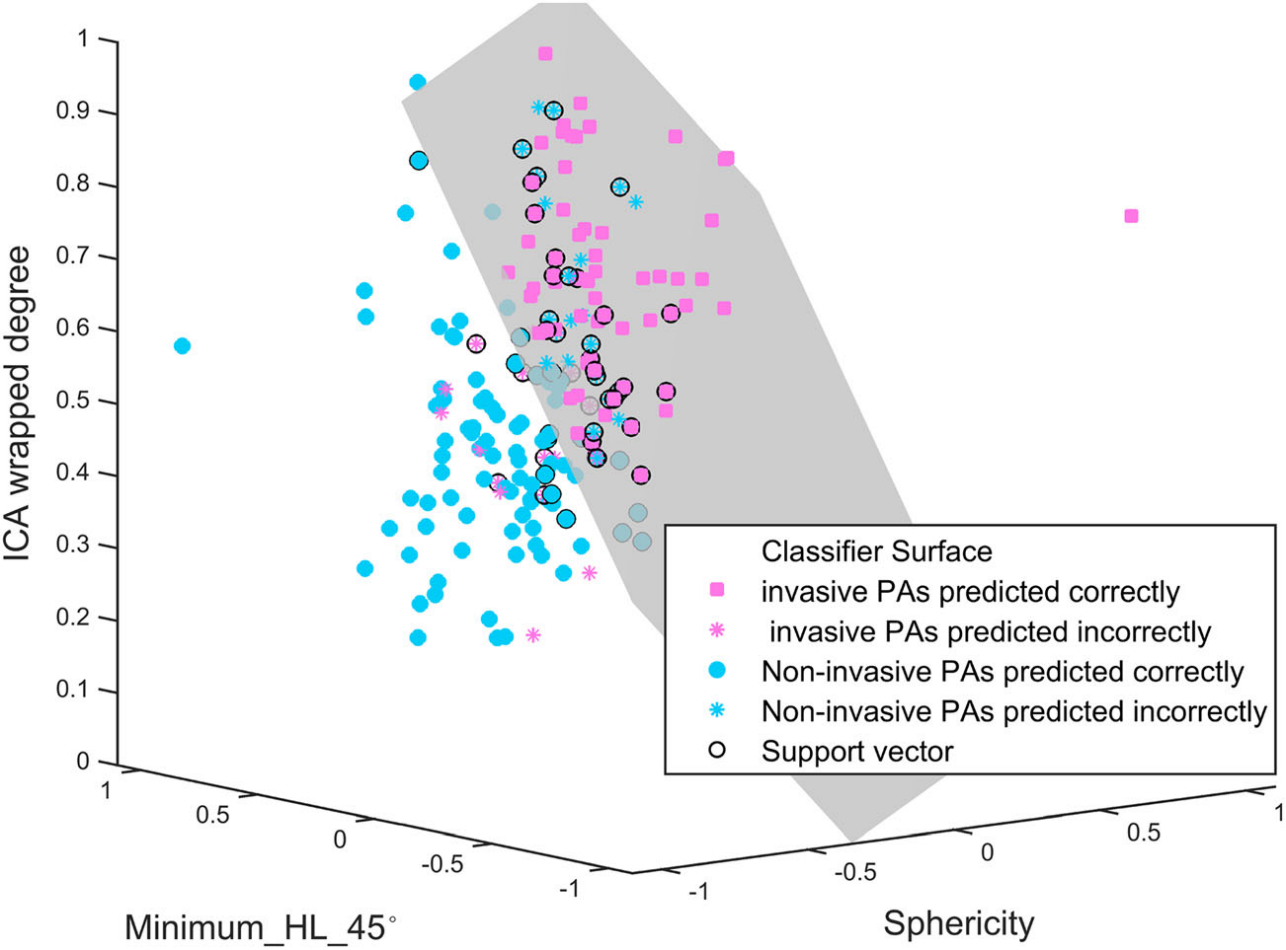
Radiomics predictive model. This model was plotted to facilitate the comprehension. The model was built based on CE-T1 MR images using linear SVMs. The x, y and z axes represent the features of Sphericity, Minimum_HL_45°, and ICA wrapped degree, respectively. These three features were normalised to the range of -1 to 1. The grey plane represents the classifier surface. The points (pink squares and sky-blue asterisks) above the classifier surface were predicted as PAs with CS invasion, while the points (sky-blue solid points and pink asterisks) below the classifier surface were predicted as PAs without CS invasion. The pink squares represent the PAs with CS invasion that were predicted correctly; the sky-blue solid points represent the PAs without CS invasion that were predicted correctly. The PAs with CS invasion, which was predicted incorrectly are shown as pink asterisks; the PAs without CS invasion, which were predicted incorrectly are shown as sky-blue asterisks. The black circles represent the support vectors calculated in the SVM model

Based on the representative features above, linear SVMs were fitted according to the training set for CE-T1, T2, and combination of CE-T1 and T2 MR images. The performances of these three models were first assessed in the training set and then validated in the completely independent test set. The CE-T1 model yielded an AUC of 0.852 in the training set and 0.826 in the test set; the T2 predictive model yielded an AUC of 0.768 in the training set and 0.733 in the test set, while the CE-T1 and T2 predictive model yielded an AUC of 0.869 in the training set and 0.803 in the test set. The accuracy, AUC, sensitivity, and specificity of these three models are shown in Table 3. The ROC curves and boxplots for CE-T1, T2, and CE-T1 and T2 MR images are plotted in Fig. 2. According to the Bayesian information criterion (BIC), the CE-T1 signature was chosen as the final radiomics signature. The formulas of these three models are shown in Supplementary S5. The CE-T1 predictive model was plotted in three-dimensional space (Fig. 3). Stratified analysis showed that our model yielded an accuracy of 76.7% in the training set and 75.6% in the test set in terms of Knosp grade 3 PAs.

**Table 3:** Performance of clinico-radiological, CE-T1, T2, and CE-T1+T2 models, and nomogram

Model	Performance	AUC (95% CI)	ACC	SEN	SPE	*p* value	Cut-off
Clinico-radiological	Training set	0.846 (0.831–0.861)	0.763	0.765	0.761	1.25E-9	0.472
	Test set	0.828 (0.812–0.844)	0.773	0.823	0.686	1.63E-8	0.472
CE-T1	Training set	0.852 (0.837–0.868)	0.753	0.851	0.660	2.33E-9	0.266
	Test set	0.826 (0.804–0.844)	**0.804**	0.800	**0.807**	1.07E-7	0.266
T2	Training set	0.768 (0.748–0.787)	0.711	0.809	0.620	5.71E-6	-0.091
	Test set	0.733 (0.712–0.754)	0.680	0.629	0.710	1.46E-4	-0.091
CE-T1+T2	Training set	0.869 (0.855–0.884)	0.753	0.851	0.660	3.80E-10	0.134
	Test set	0.803 (0.784–0.821)	0.791	0.771	0.790	8.16E-7	0.134
Nomogram	Training set Test set	0.899 (0.887–0.911) **0.871** (0.857–0.885)	0.814 0.794	0.936 **0.857**	0.700 0.758	1.31E-11 **1.51E-9**	-0.732 -0.732

The best performance in the test cohort is indicated in bold font. The cutoff values were calculated using the xtile function in R

*AUC* area under the curve, *ACC* accuracy, *SEN* sensitivity, *SPE* specificity, *CE-T1* contrast-enhanced T1 weighted MR image, *T2* T2 weighted MR image

### Clinico-radiological model development and validation

Univariate analysis was conducted in the training and test sets (Table 4**)**. Knosp grade, periarterial enhancement, and inferolateral venous compartment obliteration were significantly different between patients with CS invasion and those without CS invasion (training set: *p* < 0.001, *p* < 0.001, *p* < 0.001; test set: *p* < 0.001, *p* = 0.006, *p* = 0.012, respectively). Knosp grade, periarterial enhancement, and inferolateral venous compartment obliteration were selected for clinico-radiological model building, which yielded AUC values of 0.846 and 0.828 in the training and test sets (Table 3**)**.

**Table 4:** Univariate analysis of clinical characteristics of patients and tumours in the training set and test set

Characteristic	Training Set (*n* = 97)		*p*-value	Test Set (*n* = 97)		*p*-value
	Invasion	Non-Invasion		Invasion	Non-Invasion	
Age (yr, mean ± std)	48.09 ± 13.57	47.58 ± 11.45	0.960	44.17 ± 13.03	47.37 ± 11.93	0.293
Gender (No.)			0.1146			0.986
Male	18 (38.3%)	28 (56.0%)		18 (51.4%)	32 (51.6%)	
Female	29 (61.7%)	22 (44.0%)		17 (48.6%)	30 (48.4%)	
Knosp Grade(No.)			< 0.001			< 0.001
Grade 2	7 (14.9%)	30 (60.0%)		6 (17.1%)	46 (74.2%)	
Grade 3	40 (85.1%)	20 (40.0%)		29 (82.9%)	16 (25.8%)	
Tumour Volume (cm^3^, mean ± std)	13.79 ± 10.58	14.26 ± 21.26	0.960	14.10 ± 9.66	10.17 ± 7.94	0.053
Haemorrhage (No.)			0.064			0.051
Yes	2 (4.3%)	10 (20.0%)		1 (2.9%)	12 (19.4%)	
No	45 (95.7%)	40 (80.0%)		34 (97.1%)	50 (80.6%)	
Tumour Diameter (cm, mean ± std)	3.27 ± 0.85	3.261.07	0.960	3.38 ± 0.92	2.81 ± 0.71	0.005
Suprasellar Invasion (No.)						0.700
Yes	32 (68.1%)	32 (64.0%)		21 (60.0%)	34 (54.8%)	
No	15 (31.9%)	18 (36.0%)	0.960	14 (40.0%)	28(45.2%)	
Periarterial Enhancement (No.)			< 0.001			0.006
Yes	10 (21.3%)	33 (66.0%)		9 (25.7%)	36 (58.1%)	
No	37 (78.8%)	17 (34.0%)		26 (74.3%)	26 (41.9%)	
ICV obliteration (No.)			< 0.001			0.012
Yes	23 (48.9%)	7 (14.0%)		13 (37.1%)	8 (12.9%)	
No	24 (51.1%)	43 (86.0%)		22 (62.9%)	54 (87.1%)	

*P*-values were corrected for multiple testing by controlling the false discovery rate of 5%

*yr* year, *std* standard deviation, *ICV* inferolateral venous compartment

### Nomogram construction and validation

**Fig. 4:**
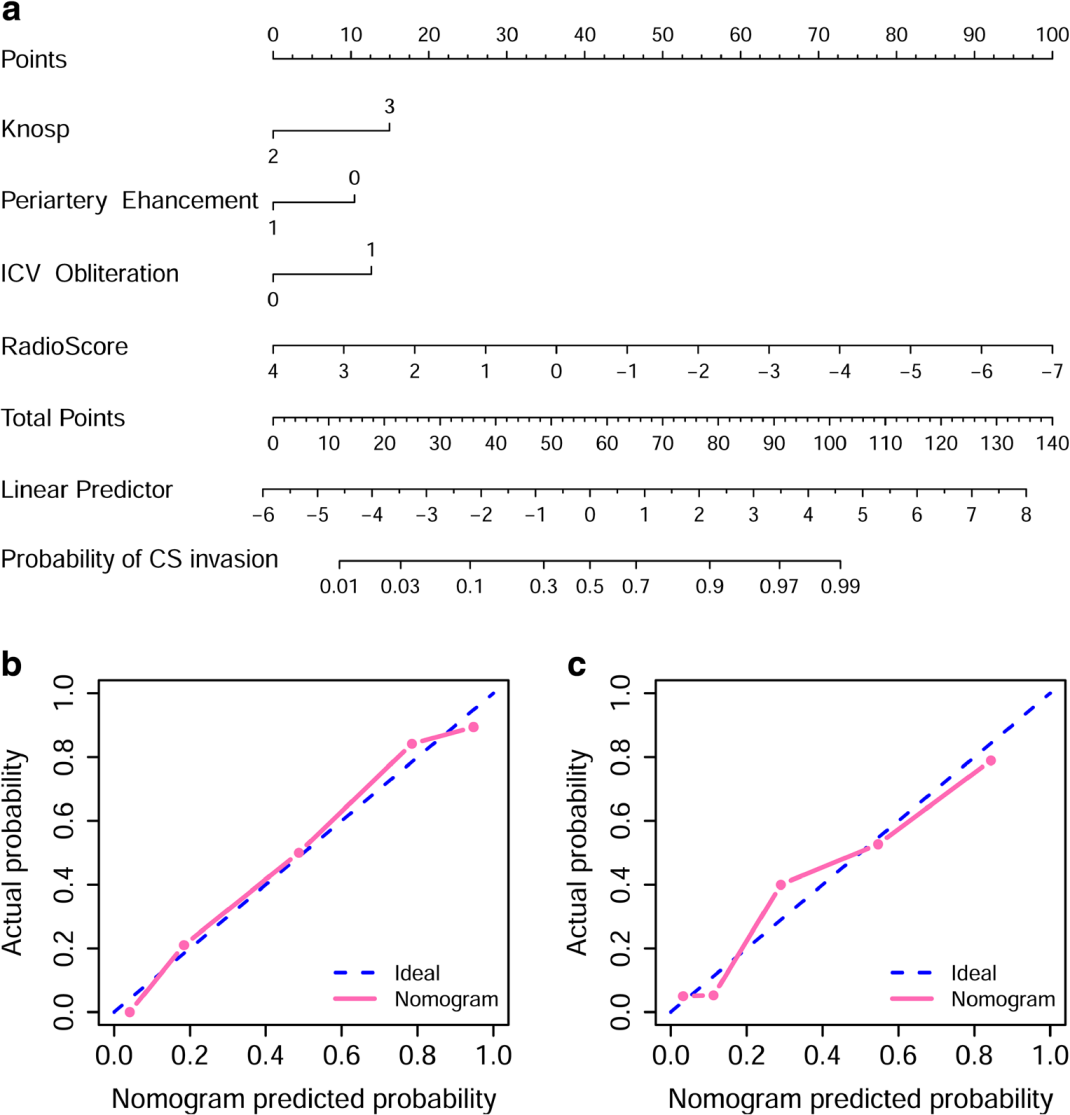
**a** A radiomics nomogram incorporating the radiomics signature, Knosp grade, periarterial enhancement, and inferolateral venous compartment obliteration on the training set. **b** Calibration curve of the radiomics nomogram on the training set. **c** Calibration curve of the radiomics nomogram on the test set. Calibration curve presents the agreement between the predicted invasion probability and observed outcomes of invasion. The diagonal blue line represents an ideal evaluation, while the black and red lines represent the performance of the nomogram. Closer fit to the diagonal blue line indicates a better evaluation

**Fig. 5:**
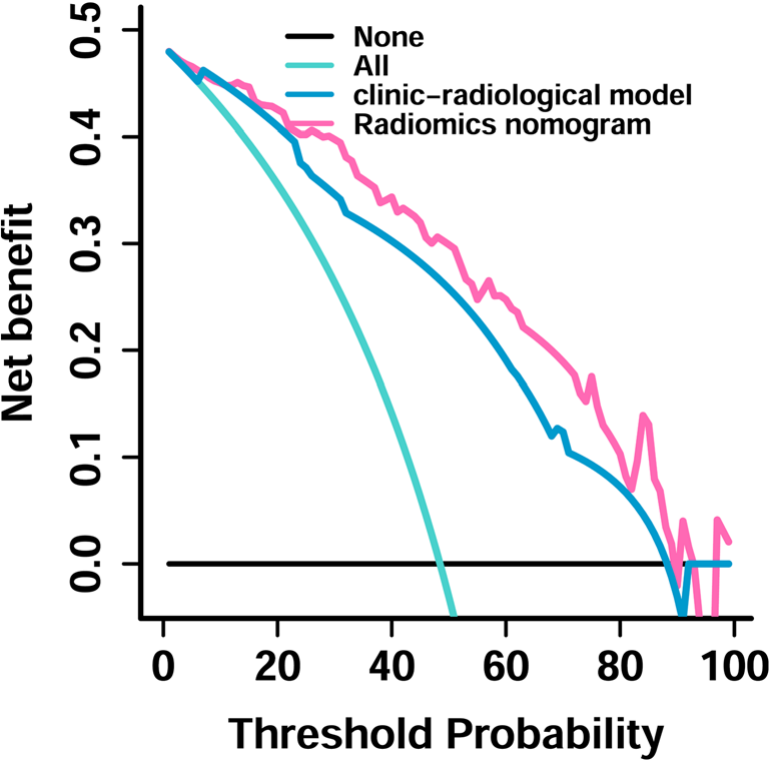
Decision curve analysis for the clinico-radiological and radiomics nomogram. The decision curve showed that if the threshold probability was higher than 20%, then using the radiomics nomogram to predict CS invasion added more benefit than either using the clinic-radiological model, treat all patients, or treat no patients

Incorporating the radiomics signature from the CE-T1 MR images, Knosp grade, periarterial enhancement, and inferolateral venous compartment obliteration, the radiomics nomogram yielded an AUC of 0.899 (95% confidence interval [CI], 0.887–0.911) in the training set and 0.871 (95% CI, 0.857-0.881) in the test set (Fig. 4 a). The radiomics nomogram significantly performed better than the clinico-radiological model (*p* = 0.021 and = 0.035 in the training and test sets, respectively; DeLong test). Furthermore, the radiomics nomogram showed a good calibration in the training and test sets (*p* = 0.664 and 0.771, respectively) (Fig. 4 b, c). The DCA for the radiomics nomogram and clinic-radiological model is shown in Fig. 5. The decision curve showed that if the threshold probability was higher than 20%, then using the radiomics nomogram to predict CS invasion by PAs added more benefit than either using the clinic-radiological model, treating all patients, or treating no patients.

## Discussion

In this study, we identified a radiomics nomogram based on CE-T1 and T2 MR images for the individualised evaluation of CS invasion in patients with PAs (Knosp grades two or three). Incorporating the radiomics signature and clinico-radiological risk factors, the nomogram outperformed the clinico-radiological and radiomics signatures in the whole sets. In the past decades, the Knosp grading has played a significant role in the evaluation of CS invasion by PAs. In their work, Knosp et al considered that invasion occurred in most Knosp grade two and all Knosp grade three PAs; however, we found that a negative CS invasion existed in both Knosp grade two and three PAs. As shown in Table 4, 85.4% of Knosp grade two PAs and 34.3% of Knosp grade three PAs were found without CS invasion. This difference in findings might be induced by the improvement of the microsurgical technology and upgrade of the microscopic and endoscopic equipment.

In this study, the CE-T1 radiomics signature was finally chosen due to its lowest BIC. This signature was fitted by three representative imaging features using linear SVM. These three features contained ICA wrapped degree, tumour sphericity, and minimum_HL_45°. ICA wrapped degree was significantly associated with CS invasion (*p* < 0.001): the higher value, the more likely the invasion could occur, which might be explained by the process of CS invasion. Most of PAs initially compress the CS and stretch its medial wall instead of invading it, which corresponds to a low value of ICA wrapped degree. With the growth of PAs, perforation of medial wall and CS invasion may occur, resulting in a high percentage of encasement of the ICA by PAs, which corresponds to a high value of ICA wrapped degree [10]. The tumour sphericity feature corresponds to the growth pattern of PAs: the lower the value of tumour sphericity, the more irregular the tumour is. Furthermore, irregular tumours can easily invade surrounding tissues [42]. We also found that the value of the feature minimum_HL_45°, which represented the grey intensity of tumour region on CE-T1 MR images, was significantly higher for PAs with CS invasion. This may be explained by the fact that invasive PAs are with abundant blood supply that could be related to the high grey intensity on CE-T1 images.

The developed nomogram incorporating the radiomics signature and clinico-radiological risk factors performed better than clinic-radiological model and radiomics models based on CE-T1, T2, and CE-T1 and T2 images. This individualised nomogram was convenient for use in pre-operative prediction of the CS invasion by Knosp grade two and three PAs for both clinicians and patients. To justify the clinical usefulness of the nomogram, DCA was conducted and showed that if patient or doctor threshold probability was > 20%, then using the nomogram to predict CS invasion added more benefit than the clinico-radiological model.

This study has several limitations. This is a single-centre study, thus requiring a multicentre validation. Additionally, in this study, tumour segmentation conducted by a senior neuro-radiologist costed plenty of time without any automatic segmentation algorithm available for PAs; thus, efficient segmentation algorithms for PAs needed to be studied. Finally, radiomics only focuses on the medical imaging of the entire tumour, where the diagnosis and prognosis of the tumour is performed using quantitative imaging features. Radiomics can be complementary to other omics such as proteomics and genomics. Therefore, it is worth looking forward that a combination of several omics would be the best choice for disease treatment.

In conclusion, this study focused on the preoperative prediction of CS invasion by Knosp grade two and three PAs and developed and validated a nomogram based on CE-T1 and T2 MR imaging. The nomogram performed better than the clinico-radiological model and might aid the surgical strategy making and allow a more focused and cost-effective follow-up and long-term management.

## Electronic supplementary material

ESM 1 (DOCX 1982 kb)

## Abbreviations

AIC: Akaike’s information criterion
AUC: Area under the curve
BIC: Bayesian information criterion
CE-T1: Contrast-enhanced T1-weighted
CI: Confidence interval
CS: Cavernous sinus
DCA: Decision curve analysis
ICA: Internal carotid artery
LASSO: Least absolute shrinkage and selection operator
PAs: Pituitary adenomas
ROC: Receiver operation characteristic
SVM: Support vector machine
