# Supplementary material for: Preoperative prediction of cavernous sinus invasion by pituitary adenomas using a radiomics method based on magnetic resonance images
Source: Eur Radiol. 2018 Sep 25;29(3):1625–34. doi: 10.1007/s00330-018-5725-3 (PMC6510860; doi:10.1007/s00330-018-5725-3)

**Supplementary S1: Inclusion and exclusion criteria**

**Inclusion criteria**: (a) pituitary adenomas confirmed by pathologic diagnosis; (b) Knosp grade 2 or 3; (c) being the first surgery; (d) pre-operative and postoperative MR imaging available; (e) clear surgical records available to judge CS invasion by PAs; (f) MR imaging performed less than 14 days before surgical resection.

**Exclusion criteria**: (a) preoperative therapy (radiotherapy, chemotherapy or chemoradiotherapy, or any drug treatment); (b) the postoperative residuals are not in agreement with surgical records.

**Supplementary S2: Acquisition parameters**

MR images were acquired in the head-first supine position on a 3-T scanner (Tim Trio, Siemens, Germany). The acquisition parameters for T2-weighted MR images were as follows: repetition time/echo time, 4500/84; flip angle, 120°; field of view, 185mm×220mm; acquisition matrix, 259×384; and slice thickness, 5 mm. The contrast enhanced T1-weighted MR images was acquired immediately after the rapid injection of contrast agent gadolinium-DTPA (0.1 mmol/kg Gadovist; Bayer Schering pharma, Berlin, Germany). The acquisition parameters for coronal and sagittal planes of contrast enhanced T1-weighted MR images were as follows: repetition time/echo time, 1200/11; flip angle, 120°; field of view, 220mm×220mm; acquisition matrix, 256×256; and slice thickness, 3 mm. The acquisition parameters for axial plane of contrast enhanced T1-weighted MR images were as follows: repetition time/echo time, 2000/9.8; flip angle, 150°; field of view, 220mm×185mm; acquisition matrix, 256×184; and slice thickness, 5 mm.

**Supplementary S3: Feature summary**

In total, 2553 features were extracted from CE-T1 images and T2-weighted MR images. Of these features, 1911 were from coronal, sagittal, and axial planes of CE-T1 MR images and 641 from T2-weighted images. All of these can be divided into four groups: (1) Tumor image intensity, (2) Tumor shape and size, (3) Tumor texture features, and (4) Tumor wavelet features. Aside from these four-group features, another feature describing the degree of ICA wrapped by PAs (ICA wrapped degree) was also calculated based on the region of ICA of the coronal planes on CE-T1 MR images. For Tumor intensity features, 11 features were extracted from sagittal, coronal and axial planes of CE-T1 MR images and T2 MR images respectively (11*3+11); For Tumor shape and size feature, 6 features from CE-T1 and T2 MR images respectively (6+6); For Texture features, there are two subgroups included, gray level co-occurrence matrix (GLCM) and gray level run length (GLRL) matrix features. In this study, voxel intensities were resampled into equally spaced bins using a bin-width of 64 Hounsfield Units. GLCM was calculated by a distance of 1 pixel in four different directions (0°, 45°, 90°, and 135°) from sagittal, coronal and axial planes of CE-T1 MR images and T2 MR images respectively, thus yielded 12 (4 directions $\times$ 3 planes) GLCM features for CE-T1 MR images and 4 (4 directions) GLCM for T2 MR images. For each GLCM, 22 imaging features were calculated, thus generated 264 (12$\times$22) GLCM features for CE-T1 MR images and 88 (4$\times$22) GLCM features for T2 MR images. GLRL matrix was calculated by a distance of 1 pixel in four different directions (0°, 45°, 90°, and 135°) from sagittal, coronal and axial planes of CE-T1 MR images and T2 MR images respectively, thus yielded 12 (4 directions $\times$ 3 planes) GLRL matrix for CE-T1 MR images and 4 (4 directions) GLRL matrix for T2 MR images. For each GLRL matrix, 7 imaging features were calculated, thus generated 84 (12$\times$7) GLRL features for CE-T1 MR images and 28 (7$\times$4) GLRL features for T2 MR images; Wavelet transform effectively decouples textural information by decomposing the original image, in a similar manner as Fourier analysis, in low and high frequencies. In this study, ‘db1’ wavelet was applied on the original MR image $X$, and $X$ was decomposed into four decompositions. Consider $L$ and $H$ to be a low-pass and high-pass function, and the wavelet decompositions of $X$ to be labeled as $X_{LL}$, $X_{LH}$, $X_{HL}$, and $X_{HH}$. Wavelet decomposition of the image is schematically depicted in **Figure S1**. For each decomposition, we computed the tumor image intensity features and the textural features. Thus, there are 1524 (3 planes $\times$ 4 decompositions $\times$ (11 intensity features + 22 GLCM features$\times$4 directions + 7 GLRL features $\times$ 4directions)) wavelet features for CE-T1 MR images and 508 (4 decompositions $\times$ (11 intensity features + 22 GLCM features $\times$ 4 directions + 7 GLRL features $\times$ 4 directions)) wavelet features for T2 MR images.

Above all, 1911 CE-T1 features, 641 T2 features, and ICA wrapped degree describing the degree of ICA wrapped by PAs were extracted (**Table S1**), and the details were as follows:

- ICA wrapped degree

The ICA wrapped degree was calculated based on the region of ICA and tumor segmented on the coronal plane of CE-T1 MR images. It was defined as the ratio of the perimeter of ICA rounded by pituitary adenomas to the perimeter of ICA, and the calculation formula was shown as following:

ICA wrapped degree = $\max\left( \frac{c_{i}}{C_{i}} \right), i=1,2,\ldots,M$,

where $M$ represented the number of the region of ICA that was depicted clearly on the coronal plane of CE-T1 MR images; $c_{i}$ represented the perimeter of $i$th ICA that was depicted clearly rounded by pituitary adenomas; $C_{i}$ represented the perimeter of $i$th ICA that was depicted clearly.

- Tumor Intensity Features

The tumor intensity features represent the distribution of voxel intensity. Let $\mathbf{X}$ represents the image matrix with $N$ voxels and **P** the histogram with $N_{l}$ discrete intensity levels. These 11 tumor intensity features are as follows:

1. Energy:

$$energy=\sum_{i}^{N} \mathbf{X}\left( i \right)^{2}$$

2. Entropy:

$$entropy=\sum_{i=1}^{N_{l}} \mathbf{P}\left( i \right)\log_{2} \mathbf{P}\left( i \right)$$

3. Kurtosis:

$$kurtosis=\frac{\frac{1}{N}\sum_{i}^{N} \left( \mathbf{X}\left( i \right)-\bar{X} \right)^{4}}{\left( \sqrt{\frac{1}{N}\sum_{i=1}^{N} \left( \mathbf{X}\left( i \right)-\bar{X} \right)^{2}} \right)^{4}}$$

Where $\bar{X}$ represents the mean of $\mathbf{X}$.

1. Maximum:

The maximum intensity value of $\mathbf{X}$.

1. Mean:

$$mean=\frac{1}{N}\sum_{i}^{N} \mathbf{X}\left( i \right)$$

2. Minimum:

The minimum intensity value of $\mathbf{X}$.

1. Root mean square(RMS)

$$RMS=\sqrt{\frac{\sum_{i}^{N} \mathbf{X}\left( i \right)^{2}}{N}}$$

2. Skewness:

$$skewness=\frac{\frac{1}{N}\sum_{i=1}^{N} \left( \mathbf{X}\left( i \right)-\bar{X} \right)^{3}}{\left( \sqrt{\frac{1}{N}\sum_{i=1}^{N} \left( \mathbf{X}\left( i \right)-\bar{X} \right)^{2}} \right)^{3}}$$

Where $\bar{X}$ represents the mean of $\mathbf{X}$.

1. Standard deviation:

$$standard deviation=\left( \frac{1}{N-1}\sum_{i=1}^{N} \left( \mathbf{X}\left( i \right)-\bar{X} \right)^{2} \right)^{\frac{1}{2}}$$

Where $\bar{X}$ represents the mean of $\mathbf{X}$.

1. Uniformity:

$$uniformity=\sum_{i=1}^{N_{l}} \mathbf{P}\left( i \right)^{2}$$

2. Variance:

$$variance=\frac{1}{N-1}\sum_{i=1}^{N} {(\mathbf{X}\left( i \right)-\bar{X})}^{2}$$

Where $\bar{X}$ represents the mean of $\mathbf{X}$.

- Shape and size features

Let V represents the volume and A the surface area of the volume of interest. These 6 radiomic features are as follows:

1. Compactness1:

$$compactness1=\frac{V}{\sqrt{\pi}A^{\frac{2}{3}}}$$

2. Compactness2:

$$compactness2=36\pi\frac{V^{2}}{A^{3}}$$

3. Sphericity:

$$sphericity=\frac{\pi^{\frac{1}{3}}{(6V)}^{\frac{2}{3}}}{A}$$

4. Surface area:

The surface area is calculated by triangulation (i.e. dividing the surface into connected triangles) and is defined as:

$$A=\sum_{i=1}^{N} \frac{1}{2}\left| \boldsymbol{a}_{i}\boldsymbol{b}_{i}\times\boldsymbol{a}_{i}\boldsymbol{c}_{i} \right|$$

Where $N$ is the total number of triangles covering the surface and $\boldsymbol{a}$**,** $\boldsymbol{b}$ and $\boldsymbol{c}$ are edge vectors of the triangles.

1. Surface to volume ratio:

$$surface to volume ratio=\frac{A}{V}$$

2. Volume:

The volume of the tumor is determined by counting the number of pixels in the tumor region and multiplying this value by the voxel size.

- Gray-level co-occurrence matrix (GLCM) features

A GLCM is defined as $\mathbf{P}\left( i,j,\delta,\alpha\right)$, a matrix with size $N_{g}\times N_{g}$­ describing the second-order joint probability function of an image, where the $\left( i,j \right)$th element represents the number of times the combination of intensity levels $i$ and $j$ occur in two pixels in the image, that are separated by a distance $\delta$ of pixels in direction $\alpha$, and $N_{g}$ is the number of discrete gray level intensities. In this study, distance $\delta$ was 1 and direction $\alpha$ was belonging to {0°, 45°, 90°, 135°}, yielding a total of 4 GLCM for each coronal, sagittal, and axial planes of CE-T1 MR images and T2 MR images respectively. Based on each GLCM, 22 textural features are generated.

Let:

$\mathbf{P}\left( i,j \right)$ be the GLCM for an arbitrary $\delta$ and $\alpha$,

$N_{g}$ be the number of discrete intensity levels in the image,

$\mu$ be the mean of $\mathbf{P}\left( i,j \right)$,

$p_{\boldsymbol{x}}\left( i \right)=\sum_{j=1}^{N_{g}} \mathbf{P}\left( i,j \right)$ be the marginal row probabilities,

$p_{\boldsymbol{y}}\left( i \right)=\sum_{i=1}^{N_{g}} \mathbf{P}\left( i,j \right)$ be the marginal column probabilities,

$\mu_{x}$ be the mean of $p_{\boldsymbol{x}}$,

$\mu_{y}$ be the mean of $p_{\boldsymbol{y}}$,

$\sigma_{x}$ be the standard deviation of $p_{\boldsymbol{x}}$,

$\sigma_{y}$ be the standard deviation of $p_{\boldsymbol{y}}$,

$p_{x+y}\left( k \right)=\sum_{i=1}^{N_{g}} \sum_{j=1}^{N_{g}} \boldsymbol{P}\left( i,j \right), i+j=k, k=2,3,\cdots,2N_{g}$,

$p_{x-y}\left( k \right)=\sum_{i=1}^{N_{g}} \sum_{j=1}^{N_{g}} \boldsymbol{P}\left( i,j \right), \left| i-j \right|=k, k=0,12,3,\cdots,N_{g}-1$,

$HX=-\sum_{i=1}^{N_{g}} p_{\boldsymbol{x}}\left( i \right){log}_{2}[p_{\boldsymbol{x}}\left( i \right)]$ be the entropy of $p_{\boldsymbol{x}}$**,**

$HY=-\sum_{i=1}^{N_{g}} p_{\boldsymbol{y}}\left( i \right){log}_{2}[p_{\boldsymbol{y}}\left( i \right)]$ be the entropy of $p_{\boldsymbol{y}}$**,**

$H=-\sum_{i=1}^{N_{g}} \sum_{j=1}^{N_{g}} \boldsymbol{P}\left( i,j \right){log}_{2}[\boldsymbol{P}\left( i,j \right)]$ be the entropy of $\mathbf{P}(i,j)$**,**

$HXY1=-\sum_{i=1}^{N_{g}} \sum_{j=1}^{N_{g}} \boldsymbol{P}\left( i,j \right){log}_{2}[p_{\boldsymbol{x}}\left( i \right)p_{\boldsymbol{y}}\left( i \right)]$,

$HXY2=-\sum_{i=1}^{N_{g}} \sum_{j=1}^{N_{g}} p_{\boldsymbol{x}}\left( i \right)p_{\boldsymbol{y}}\left( i \right){log}_{2}[p_{\boldsymbol{x}}\left( i \right)p_{\boldsymbol{y}}\left( i \right)]$,

1. Autocorrelation:

$$autocorrelation=\sum_{i=1}^{N_{g}} \sum_{j=1}^{N_{g}} ij\mathbf{P}\left( i,j \right)$$

2. Cluster Prominence:

$$cluster prominence=\sum_{i=1}^{N_{g}} \sum_{j=1}^{N_{g}} \left[ i+j-\mu_{x}\left( i \right)-\mu_{y}(j) \right]^{4}\mathbf{P}\left( i,j \right)$$

3. Cluster Shade:

$$cluster shade=\sum_{i=1}^{N_{g}} \sum_{j=1}^{N_{g}} \left[ i+j-\mu_{x}\left( i \right)-\mu_{y}(j) \right]^{3}\mathbf{P}\left( i,j \right)$$

4. Cluster Tendency:

$$cluster tendency=\sum_{i=1}^{N_{g}} \sum_{j=1}^{N_{g}} \left[ i+j-\mu_{x}\left( i \right)-\mu_{y}(j) \right]^{2}\mathbf{P}\left( i,j \right)$$

5. Contrast:

$$contrast=\sum_{i=1}^{N_{g}} \sum_{j=1}^{N_{g}} \left| i-j \right|^{2}\mathbf{P}\left( i,j \right)$$

6. Correlation:

$$correlation=\frac{\sum_{i=1}^{N_{g}} \sum_{j=1}^{N_{g}} ij\mathbf{P}\left( i,j \right)-\mu_{x}(i)\mu_{y}(j)}{\sigma_{x}(i)\sigma_{y}(j)}$$

7. Difference entropy:

$$difference entropy=-\sum_{i=1}^{N_{g}-1} \mathbf{P}_{\boldsymbol{x-y}}\left( i \right){log}_{2}[\mathbf{P}_{\boldsymbol{x-y}}\left( i \right)]$$

8. Dissimilarity:

$$dissimilarity=\sum_{i=1}^{N_{g}} \sum_{j=1}^{N_{g}} \left| i-j \right|\mathbf{P}\left( i,j \right)$$

9. Energy:

$$energy=\sum_{i=1}^{N_{g}} \sum_{j=1}^{N_{g}} {[\mathbf{P}\left( i,j \right)]}^{2}$$

10. Entropy:

$$entropy=-\sum_{i=1}^{N_{g}} \sum_{j=1}^{N_{g}} \boldsymbol{P}\left( i,j \right){log}_{2}[\boldsymbol{P}\left( i,j \right)]$$

11. Homogeneity1:

$$homogeneity1=\sum_{i=1}^{N_{g}} \sum_{j=1}^{N_{g}} \frac{\mathbf{P}\left( i,j \right)}{1\boldsymbol{+}\left| \boldsymbol{i-j} \right|}$$

12. Homogeneity2:

$$homogeneity2=\sum_{i=1}^{N_{g}} \sum_{j=1}^{N_{g}} \frac{\mathbf{P}\left( i,j \right)}{1\boldsymbol{+}\left| \boldsymbol{i-j} \right|^{\boldsymbol{2}}}$$

13. Informational measure of correlation 1 (IMC1):

$$IMC1=\frac{HXY-HXY1}{max\{HX,HY\}}$$

14. Informational measure of correlation 2 (IMC2):

$$IMC2=\sqrt{1-e^{1-(HXY2-HXY)}}$$

15. Inverse Difference Moment Normalized (IDMN):

$$IDMN=\sum_{i=1}^{N_{g}} \sum_{j=1}^{N_{g}} \frac{\mathbf{P}\left( i,j \right)}{1\boldsymbol{+}\frac{\left| \boldsymbol{i-j} \right|^{\boldsymbol{2}}}{\boldsymbol{N}^{\boldsymbol{2}}}}$$

16. Inverse Difference Normalized (IDN):

$$IDN=\sum_{i=1}^{N_{g}} \sum_{j=1}^{N_{g}} \frac{\mathbf{P}\left( i,j \right)}{1\boldsymbol{+}\frac{\left| \boldsymbol{i-j} \right|}{\boldsymbol{N}}}$$

17. Inverse variance:

$$inverse variance=\sum_{i=1}^{N_{g}} \sum_{j=1}^{N_{g}} \frac{\mathbf{P}\left( i,j \right)}{\left| \boldsymbol{i-j} \right|^{\boldsymbol{2}}}, i\neq j$$

18. Maximum probability:

$$maxmium probability=max\{\mathbf{P}\left( i,j \right)\}$$

19. Sum average:

$$sum average=\sum_{i=2}^{2N_{g}} i\mathbf{P}_{x+y}(i\boldsymbol{)}$$

20. Sum entropy:

$$sum entropy=-\sum_{i=2}^{2N_{g}} \mathbf{P}_{\boldsymbol{x+y}}\left( i \right){log}_{2}[\mathbf{P}_{\boldsymbol{x+y}}\left( i \right)]$$

21. Sum variance:

$$sum variance=\sum_{i=2}^{2N_{g}} \left( i-SE \right)^{2}\mathbf{P}_{\boldsymbol{x+y}}\left( i \right)$$

22. Variance:

$$Variance=\sum_{i=1}^{N_{g}} \sum_{j=1}^{N_{g}} \left( i-\mu\right)^{2}\mathbf{P}_{\boldsymbol{x+y}}\left( i \right)$$

- Gray-level run-length matrix (GLRL) features

Run length metrics quantify gray level runs in an image. A gray level run is defined as the length in number of pixels, of consecutive pixels that have the same gray level value. In a GLRL $p\left( i,j|\theta\right)$, the $(i,j)$th element describes the number of times $j$ a gray level $i$ appears consecutively in the direction specified by $\theta$, and $N_{g}$ is the number of discrete gray level intensities. In this study, a GLRL was computed for each direction in set {0°, 45°, 90°, 135°}, from which the below textural features were derived.

Let:

$p\left( i,j|\theta\right)$ be the $\left( i,j \right)$th entry in the given run-length matrix $p$ for a direction $\theta$,

$N_{g}$ the number of discrete intensity values in the image,

$N_{r}$ the number of different run lengths,

$N_{p}$ the number of voxels in the image.

1. Short run emphasis (SRE):

$$SRE=\frac{\sum_{i=1}^{N_{g}} \sum_{j=1}^{N_{r}} [\frac{p(i,j|\theta)}{j^{2}}]}{\sum_{i=1}^{N_{g}} \sum_{j=1}^{N_{r}} p(i,j|\theta)}$$

2. Long run emphasis (LRE):

$$LRE=\frac{\sum_{i=1}^{N_{g}} \sum_{j=1}^{N_{r}} j^{2}p(i,j|\theta)}{\sum_{i=1}^{N_{g}} \sum_{j=1}^{N_{r}} p(i,j|\theta)}$$

3. Gray level non-uniformity (GLN):

$$GLN=\frac{\sum_{i=1}^{N_{g}} {[\sum_{j=1}^{N_{r}} p\left( i,j | \theta\right)]}^{2}}{\sum_{i=1}^{N_{g}} \sum_{j=1}^{N_{r}} p(i,j|\theta)}$$

4. Run length non-uniformity (RLN):

$$RLN=\frac{\sum_{i=1}^{N_{r}} {[\sum_{j=1}^{N_{g}} p\left( i,j | \theta\right)]}^{2}}{\sum_{i=1}^{N_{g}} \sum_{j=1}^{N_{r}} p(i,j|\theta)}$$

5. Run percentage (RP):

$$RP=\sum_{i=1}^{N_{g}} \sum_{j=1}^{N_{r}} \frac{p(i,j|\theta)}{N_{p}}$$

6. Low gray level rum emphasis (LGLRE):

$$LGLRE=\frac{\sum_{i=1}^{N_{g}} \sum_{j=1}^{N_{r}} [\frac{p(i,j|\theta)}{i^{2}}]}{\sum_{i=1}^{N_{g}} \sum_{j=1}^{N_{r}} p(i,j|\theta)}$$

7. High gray level rum emphasis (HGLRE):

$$HGLRE=\frac{\sum_{i=1}^{N_{g}} \sum_{j=1}^{N_{r}} i^{2}p(i,j|\theta)}{\sum_{i=1}^{N_{g}} \sum_{j=1}^{N_{r}} p(i,j|\theta)}$$

**
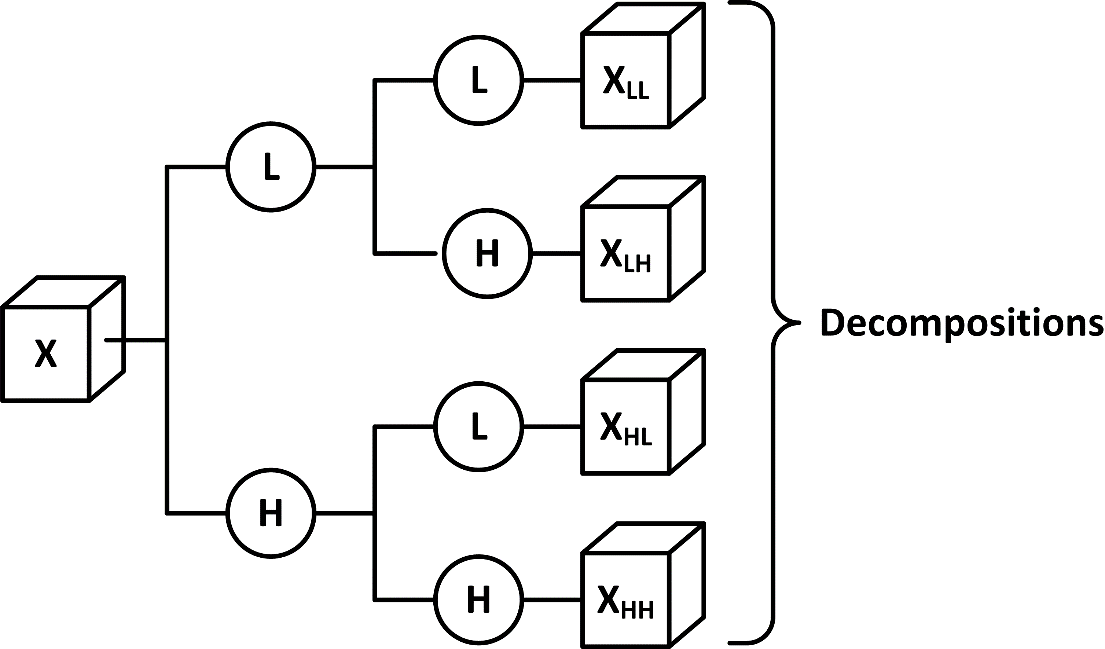
**

**Figure S1.** Schematic of two-dimensional wavelet transform applied to each MR images. The original image $X$ is decomposed into 4 decompositions: $X_{LL}$, $X_{LH}$, $X_{HL}$ and $X_{HH}$.

**Table S1**. The summary of the features extracted.

| Feature Group | Feature Names |
| --- | --- |
| Tumor Intensity | Maximum; Minimum; Mean; Standard Variation; Variance;  Root Mean Square; Skewness; Kurtosis; Uniformity; Energy;  Entropy. |
| Shape and Size | Volume; Surface Area; Compactness 1; Compactness 2;  Surface to Volume Ratio; Sphericity. |
| Texture Features | GLCM_Contrast; GLCM_Correlation 1; GLCM_Correlation 2;  GLCM_Variance; GLCM_Sum Average; GLCM_Sum Variance;  GLCM_Sum Entropy; GLCM_Entropy; GLCM_Homogeneity 1;  GLCM_Homogeneity 2; GLCM_Difference Variance;  GLCM_Information Measure of Correlation 1;  GLCM_Information Measure of Correlation 2;  GLCM_Inverse Difference Normalized; GLCM_Difference Entropy;  GLCM_Inverse Difference Moment Normalized;  GLCM_Autocorrelation; GLCM_Cluster Prominence;  GLCM_Cluster Shade; GLCM_Dissimilarity;  GLCM_Energy; GLCM_Maximum Probability;  GLRL_Short Run Emphasis; GLRL_Long Run Emphasis;  GLRL_Gray Level Non-Uniformity; GLRL_Run Length Non-Uniformity;  GLRL_Run Percentage; GLRL_Low Gray Level Run Emphasis;  GLRL_High Gray Level Run Emphasis; |
| Wavelet Features | Extract tumor intensity features and texture features for each decomposition. |

**Supplementary S4: Feature Selection**

LASSO is a powerful method for many classification problem, particularly ones with many features. In this study, we used the LASSO to selected the most important features from the training set $\left\{ \left( x^{\left( i \right)},y^{\left( i \right)} \right),i=1,\ldots,M \right\}$. Here, each $x^{\left( i \right)}\in\mathbb{R}^{N}$ is an $N$-dimensional feature vector, and $y^{\left( i \right)}\in\left\{ 0,1 \right\}$ is a class label. The LASSO minimizes a log partial likelihood subject to the sum of the absolute values of the parameters being bounded by a constant $C$:

$\min_{\theta} \sum_{i=1}^{M} -\log p\left( y^{\left( i \right)} | x^{\left( i \right)};\theta\right)$, subject to $\left\| \theta\right\|_{1}\leq C$.

Here $p\left( y=1 | \mathbf{x};\theta\right)=1-p\left( y=0 | \mathbf{x};\theta\right)=\frac{1}{1+exp(-\theta^{T}\mathbf{x})}$, $\theta\in\mathbb{R}^{N}$ are the coefficients to be optimized. Benefit from the absolute constraint, LASSO could select the most important features by shrinking the coefficients of useless features to zero. In this study, the constant $C$­ was determined using five-fold cross-validation based on 1-SE criteria. In this study, the LASSO was conducted using “lasso” function in MATLAB 2012a.

In the phrase of feature selection, three representative features were selected from CE-T1 MR images; one from T2 MR images; four from both CE-T1 MR images and T2 MR images (**Figure S2)**.

A

C

E

B

D

F

**Figure S2** Feature selection using the LASSO binary logistic regression model.

(A): Tuning parameter ($\lambda$) selection in the LASSO model used five-fold cross-validation via minimum criteria based on CE-T1 MR images. The mean squared error was plotted versus $log(\lambda)$. The dotted vertical lines were drawn at the optimal values using the minimum criteria and the 1-SE criteria. A value $\lambda$ of 0.180 with $\log\left( \lambda\right)=-1.714$ was chosen (1-SE criteria) according to five-fold cross-validation. (B): LASSO coefficient profiles of the 65 CE-T1 MR imaging features with low redundancy. The optimal $\lambda$ the same as which in (A) resulted in 2 non-zero coefficients.

(C) Tuning parameter ($\lambda$) selection in the LASSO model used five-fold cross-validation via minimum criteria based on T2 MR images. The mean squared error was plotted versus $log(\lambda)$. The dotted vertical lines were drawn at the optimal values using the minimum criteria and the 1-SE criteria. A value $\lambda$ of 0.170 with $\log\left( \lambda\right)=-1.770$ was chosen (1-SE criteria) according to five-fold cross-validation. (D): LASSO coefficient profiles of the 24 T2 MR imaging features with low redundancy. The optimal $\lambda$ the same as which in (C) resulted in one non-zero coefficient.

(E) Tuning parameter ($\lambda$) selection in the LASSO model used five-fold cross-validation via minimum criteria based on both CE-T1 and T2 MR images. The mean squared error was plotted versus $log(\lambda)$. The dotted vertical lines were drawn at the optimal values using the minimum criteria and the 1-SE criteria. A value $\lambda$ of 0.198 with $\log\left( \lambda\right)=-1.621$ was chosen (1-SE criteria) according to five-fold cross-validation. (F): LASSO coefficient profiles of the 89 CE-T1 and T2 MR imaging features with low redundancy. The optimal $\lambda$ the same as which in (E) resulted in 3 non-zero coefficients.

**Supplementary S5: Radiomics score calculation formula:**

$$Radiomics Score=-1\times b+\sum_{i=1}^{N} c_{i}\times\boldsymbol{x'}\boldsymbol{sv}_{\boldsymbol{i}}$$

Where

b is the intercept,

N the number of support vectors,

$\boldsymbol{sv}_{\boldsymbol{i}}$ the $i$th support vector,

$\boldsymbol{x}$ the new data. For CE-T1 MR images, $\boldsymbol{x}\mathbf{=[}Sphericity\_T1,minmium\_HL\_T1,DIACE\_T1\mathbf{]'}$; For T2 MR images, $\boldsymbol{x}\mathbf{=[}Sphericity\_T2\mathbf{]}$**;** For both CE-T1 and T2 MR images, $\boldsymbol{x}\mathbf{=[}Sphericity\_T1,minmium\_HL\_T1,LGLRE\_HH\_135^{\circ}\_T2,DIACE\_T1\mathbf{]'}$. These three features were from CE-T1 MR images and were described in **Supplementary S3**,

$c_{i}$ representing the trade-off between misclassification of training examples and simplicity of the decision surface.

**Figure S3.** CE-T1 and T2 MR images of pituitary adenomas. The region of tumor was contoured by green curves on both CE-T1 and T2 MR images; the region of left and right ICA was contoured by blue and red curves on the coronal plane of CE-T1 MR images, respecticely.(A1) and (A2) represent the CE-T1 and T2 MR image of Knosp grade two pituitary adenomas without cavernous sinus invasion; (B1) and (B2) represent the CE-T1 and T2 MR images of Knosp grade two pituitary adenomas with cavernous sinus invasion; (C1) and (C2) represent the CE-T1 and T2 MR image of Knosp grade three pituitary adenomas without cavernous sinus invasion; (D1) and (D2) represent the CE-T1 and T2 MR image of Knosp grade three pituitary adenomas with cavernous sinus invasion.


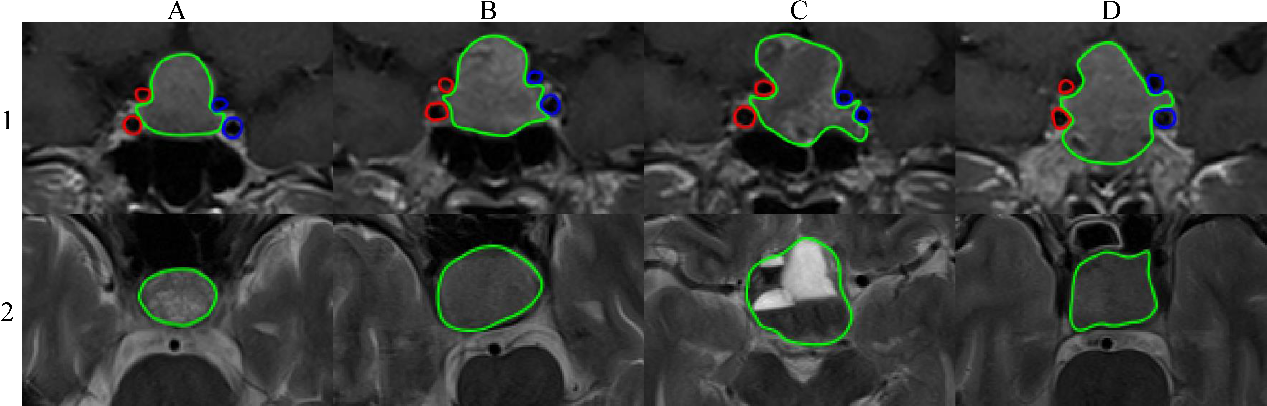

Supplement: Supplementary file 1 — (DOCX 1982 kb) [file 330_2018_5725_MOESM1_ESM.docx]
